# Association of Initiation of Dialysis With Hospital Length of Stay and Intensity of Care in Older Adults With Kidney Failure

**DOI:** 10.1001/jamanetworkopen.2020.0222

**Published:** 2020-02-28

**Authors:** Helen Tam-Tham, Pietro Ravani, Jianguo Zhang, Robert G. Weaver, Robert R. Quinn, Matthew T. James, Ping Liu, Braden J. Manns, Marcello Tonelli, Paul E. Ronksley, Tyrone G. Harrison, Chandra Thomas, Sara Davison, Brenda R. Hemmelgarn

**Affiliations:** 1 Department of Medicine, Cumming School of Medicine, University of Calgary, Calgary, Alberta, Canada; 2 Community Health Sciences, Cumming School of Medicine, University of Calgary, Calgary, Alberta, Canada; 3 Department of Medicine, Faculty of Medicine & Dentistry, University of Alberta, Edmonton, Alberta, Canada

## Abstract

**Importance:**

Comparative outcome data examining the association of dialysis initiation with hospital length of stay and intensity of care in older adults with kidney failure are scarce, and prior studies are limited to patients treated by nephrology teams.

**Objective:**

To compare in-hospital days and intensity of care among older adults with kidney failure who were treated vs not treated with maintenance dialysis.

**Design, Setting, and Participants:**

This population-based, retrospective cohort study included adults in Alberta, Canada, 65 years or older with kidney failure, defined by at least 2 consecutive outpatient estimated glomerular filtration rate values of less than 10 mL/min/1.73 m^2^ spanning a period of at least 90 days from May 15, 2002, to March 31, 2014. Data were analyzed from August 1, 2017, to August 29, 2019.

**Exposures:**

Time-varying exposure to maintenance dialysis for treatment of kidney failure.

**Main Outcomes and Measures:**

The primary outcome was rate of in-hospital days. Secondary outcomes included rates of hospital admissions, intensive care unit admissions, cardiopulmonary resuscitations, inpatient palliative care, and emergency department visits; risk of in-hospital death; and time to admission to long-term care.

**Results:**

A total of 968 patients (median age, 78.5 [interquartile range, 72.4-84.7] years; 489 men [50.5%]; median follow-up, 2.0 [interquartile range, 0.8-3.9] years) were included in the analysis. Patients who underwent dialysis spent more adjusted in-hospital days per person-year (36.25 [95% CI, 30.72-41.77] vs 14.65 [95% CI, 12.28-17.02]; incidence rate ratio [IRR], 2.47 [95% CI, 1.99-3.08]). However, the dialysis group did not have a higher rate of hospital admissions (1.18 [95% CI 1.07-1.29] vs 1.32 [95% CI 1.17-1.48] per year; IRR, 0.89 [95% CI, 0.77-1.03]). Patients in the dialysis group had a higher rate of intensive care unit admissions per 1000 hospitalizations (98.37 [95% CI, 81.09-115.65] vs 54.51 [95% CI, 37.76-71.26]; IRR, 1.80 [95% CI, 1.28-2.54]) and lower rates of inpatient palliative care per 1000 in-hospital days (3.92 [95% CI, 3.13-4.72] vs 8.60 [95% CI, 6.3-11.0]; IRR, 0.45 [95% CI, 0.32-0.64]).

**Conclusions and Relevance:**

In this cohort study, compared with nondialysis care, patients who received maintenance dialysis spent more time in the hospital and were more likely to be admitted to intensive care units. This finding suggests trade-offs between longer survival and higher intensity of use of health care services as a function of dialysis initiation. Maintenance dialysis may be a proxy for the type of philosophy of care driving increased in-hospital time and intensive care and less use of palliative care.

## Key Points

**Question:**

Is maintenance dialysis associated with increased time in the hospital and more intensive care in older adults with kidney failure?

**Findings:**

In this cohort study of 968 older adults with kidney failure, those who received dialysis spent a mean of 22 more in-hospital days each year and were almost 2 times more likely to receive intensive care than those who did not receive dialysis.

**Meaning:**

These findings suggest that maintenance dialysis is associated with increased length of hospital stay and intensive care and less use of palliative care.

## Teaser

This cohort study compares in-hospital days and intensity of care among older adults with kidney failure who were treated vs not treated with maintenance dialysis.

## Introduction

Older adults with kidney failure may base their decision to initiate dialysis on factors such as survival and time spent in the hospital.^1,2^ In a recent study, Tam-Tham et al^3^ found that mortality was lower among older adults receiving maintenance dialysis than in those who received nondialysis care, although this benefit was observed only in the first 3 years after kidney failure (defined by an estimated glomerular filtration rate [eGFR] of <10 mL/min/1.73 m^2^). The number of hospital-free days has been emphasized as an important patient-oriented outcome, because it provides insight to quality of life for patients making a treatment decision to initiate dialysis or not.^4,5^

Comparative data on these outcomes have been limited.^6^ Furthermore, given that dialysis can be an option for patients not followed up by nephrology, prior studies^6^ comparing outcomes between dialysis and nondialysis care have been restricted to patients treated by nephrology teams with a potential for lead time and immortal time biases. Lead time bias from residual kidney function will overestimate risk time of outcomes for patients with more lead time (eg, patients receiving nondialysis treatment with higher eGFR and without clinical indications for dialysis will have more risk time than patients receiving nondialysis treatment with lower eGFR). Immortal time bias will overestimate risk time of outcomes for patients in dialysis vs nondialysis groups.

Hence, we sought to compare days spent in the hospital and intensity of care in a population-based cohort of older adults treated or not treated with maintenance dialysis, addressing design issues including exposure and outcome definitions to minimize common forms of bias and inform future randomized clinical trials in this area. Our primary objective was to compare the number of in-hospital days as a direct measure of hospital-free survival days. Our secondary objectives were to compare rates of hospital admissions, rates of admission to intensive care units (ICUs), rates of cardiopulmonary resuscitation, rates of inpatient palliative care, risk of in-hospital deaths, rates of emergency department visits, and time to admission to long-term care.

## Methods

### Patient Population

We used unique Alberta Personal Health Numbers to link provincial administrative and laboratory data to assemble a cohort of Alberta, Canada, residents 65 years or older and identified as having kidney failure from May 15, 2002, to March 31, 2014. As described previously,^3^ we defined kidney failure as at least 2 consecutive outpatient eGFR measurements of less than 10 mL/min/1.73 m^2^, calculated using the CKD-EPI (Chronic Kidney Disease Epidemiology Collaboration) equation,^7^ during a period of at least 90 days. The first eGFR after the 90-day period was used to define the index date (ie, start of follow-up) for patients in the nondialysis group. We excluded patients who died on their index date as well as those treated with maintenance dialysis or kidney transplant before or on the index date. Ethics approval and waiver of patient consent were granted from the Conjoint Health Research Ethics Review Board at the University of Calgary, Calgary, Alberta, for the retrospective use of deidentified data. This study followed the Strengthening the Reporting of Observational Studies in Epidemiology (STROBE) reporting guideline.

### Study Design

We used a retrospective cohort design with population-based clinical data from Alberta.^8^ To minimize immortal time bias, we used a time-varying exposure variable to characterize treatment status during follow-up.^9^ To minimize lead time bias, we set a 90-day period of at least 2 consecutive outpatient eGFR measurements of less than 10 mL/min/1.73 m^2^ to reflect a level of kidney function at which patients and their physicians would have made a decision whether to pursue maintenance dialysis or not. To minimize treatment-selection bias, we controlled for measured confounding by regression analysis.

### Exposure

The exposure of interest was treatment with maintenance dialysis. We defined incident maintenance dialysis cases (hemodialysis or peritoneal dialysis) from dialysis registries in Alberta.^10^ We excluded periods of dialysis lasting less than 90 days followed by recovery of kidney function and included patients if they died within 90 days and the intent of the treatment (established by review of electronic medical records) was maintenance dialysis. A priori, we elected to use a time-varying exposure variable to characterize treatment status during follow-up.

### Outcomes

#### Hospitalizations

The primary outcome was rate of cumulative days spent in the hospital, measured as the number of patient in-hospital days per person-year. We deemed patients at risk from the index date to study end date (March 31, 2015) or the date of kidney transplantation, death, or outmigration from the province, whichever was earliest. The study end date was chosen to allow for at least 1 year of follow-up for all patients.

### Secondary Outcomes

Secondary outcomes were rates of hospital admissions and intensity of care for patients hospitalized as defined by rates of admission to the ICU, receipt of inpatient cardiopulmonary resuscitation, receipt of inpatient palliative care consultations (eTable 1 in the Supplement), and risk of in-hospital death. We used the Hospital Discharge Abstracts database for inpatient cumulative length of stay and number of hospital admissions. We determined death dates from the Alberta Health Registry and from Vital Statistics; we defined in-hospital deaths as death occurring on the date of hospital discharge.

We examined rate of emergency department visits using the Ambulatory Care data.^8^ We examined risk of long-term care admission using delivery site type information from physician claims, supplemented by discharge disposition to a continuing care facility from the Hospital Discharge Database.^11^

### Covariates

We identified baseline characteristics at the index date. We identified demographic characteristics from the Alberta Health Registry file, including age and sex. We used the Canadian Census with the Statistics Canada Postal Code Conversion File to determine rural location of residence, as defined previously.^3^

We identified diabetes^12^ and hypertension^13^ from hospital discharge records and physician claims using validated algorithms. We identified other comorbidities based on the Charlson-Deyo comorbidity index (dementia, cerebrovascular disease, myocardial infarction, congestive heart failure, peripheral vascular disease, chronic obstructive pulmonary disease, mild liver disease, moderate and severe liver disease, peptic ulcer disease, rheumatologic disease, paraplegia or hemiplegia, and cancer) using validated *International Classification of Diseases, Ninth Revision,* and *International Statistical Classification of Diseases and Related Health Problems, Tenth Revision*, coding algorithms from physician claims and hospitalization data, respectively.^14,15^ We identified comorbidities by at least 1 hospital diagnostic code or 2 physician claims codes in the 3 years before cohort entry.

Receipt of angiotensin-converting enzyme inhibitors, angiotensin-receptor blockers, and statin use was defined by at least 1 prescription dispensed for these medications within the year before the index date according to the Alberta Health Blue Cross drug file. We used the most recent outpatient albuminuria measurement within 2 years before the index date. Albuminuria was categorized in accordance with international guidelines as normal/mild, moderate, severe, or unmeasured with the following types of measurement in descending order of preference: ratio of albumin to creatinine levels (<3, 3-30, and >30 mg/mmol or <30, 30-300, and >300 mg/g), ratio of protein to creatinine levels (<15, 15-50, and >50 mg/mmol or <150, 150-500, and >500 mg/g), and urine dipstick findings (negative or trace, +1, or +2 or greater).^16^ Rapid progression of eGFR was defined as a decline of greater than 5 mL/min/1.73 m^2^ per year based on eGFR values within 3 years before the index date.^16,17^

### Statistical Analysis

Data were analyzed from August 1, 2017, to August 29, 2019. For the primary analysis, we first examined the rate and incidence rate ratio (IRR) of cumulative length of in-hospital stay (days) per year among those treated vs not treated with maintenance dialysis. As reported in previous work,^18^ we found that length of stay data were overdispersed. After graphical and goodness-of-fit tests, we used negative binomial regression to examine the exposure-outcome association. For primary and secondary analyses, we adjusted for potential confounders, including sex, age, rural/urban location of residence, residence in a long-term care facility, prior hospitalization, index eGFR, progression of eGFR per year, angiotensin-converting enzyme inhibitor/angiotensin-receptor blocker use, statin use, comorbidities as defined above, and days from the first qualifying to the index eGFR. We estimated adjusted rates at means of covariates. For secondary analyses, we used negative binomial regression to examine rate of hospital admissions and rate of emergency department visits, accounting for patients with multiple events. Time at risk of new hospital admissions and emergency department visits excluded days already in the hospital. We used Cox proportional hazards regression to examine risk of long-term care admission and modified Poisson regression for risk of in-hospital death (compared with death not in a hospital setting).^19^ We assessed model validity using graphical methods (eg, distributions of emergency department visits and number of hospital admissions) and checked model assumptions (eg, the proportional hazards assumption for Cox regression assessed graphically and with Schoenfeld residuals for risk of long-term care admission). When examining the IRR of cumulative length of stay and hospital admissions, we conducted 2 sensitivity analyses excluding inpatient dialysis initiation and patients with prior hospitalization, because these patients may have a poorer health status than patients initiating dialysis in the outpatient setting or lacking prior hospitalization. We conducted statistical analyses with Stata software, version 14 (StataCorp LLC).

## Results

### Patient Characteristics

**Figure 1.**
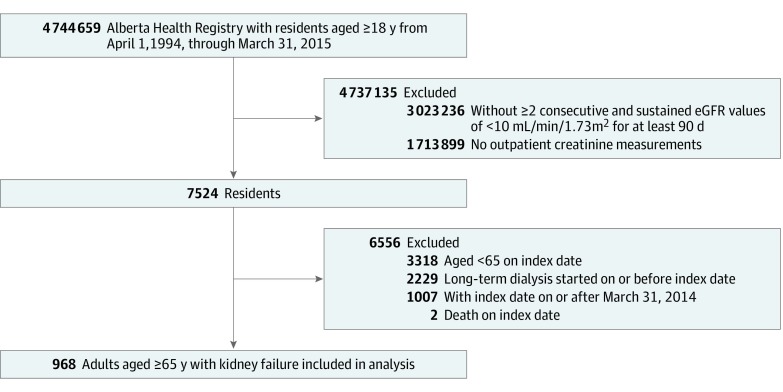
Cohort Formation eGFR indicates estimated glomerular filtration rate.

A total of 968 patients met the cohort inclusion criteria (median age, 78.5 [interquartile range {IQR}, 72.4-84.7] years; 489 men [50.5%] and 479 women [49.5%]; median eGFR, 8.0 [IQR, 7.0-8.9] mL/min/1.73 m^2^) (Figure 1); of these, 557 (57.5%) received maintenance dialysis and 411 (42.5%) did not (Table). Overall, patients not treated with dialysis were more likely to be female (237 of 411 [57.7%]), to be older (median age, 83.6 [IQR, 78.3-88.2] years), to have greater comorbidity scores (94 of 411 [22.9%] with Charlson-Deyo comorbidity index score ≥7), and to reside in a long-term care facility (95 of 411 [23.1%]). The overall median follow-up was 2.0 (IQR, 0.8-3.9) years; among patients in the dialysis group, median time from index date to dialysis initiation was 0.3 (IQR, 0.1-0.7) years.

**Table.** Baseline Patient Characteristics

Characteristic	Dialysis Group, No. (%)	
	Treated (n = 557)	Not Treated (n = 411)
Male	315 (56.6)	174 (42.3)
Female	242 (43.4)	237 (57.7)
Age, median (IQR), y	75.2 (70.5-80.2)	83.6 (78.3-88.2)
Rural location of residence	101 (18.1)	57 (13.9)
Residing in a long-term care facility	35 (6.3)	95 (23.1)
Hospitalization 1 y before index date	186 (33.4)	187 (45.5)
eGFR at index date, median (IQR), mL/min/1.73 m^2^	7.9 (7.0-8.8)	8.1 (6.9-9.0)
Rapid decline of eGFR (>5 mL/min/1.73 m^2^) per y in 3 y before index date	252 (45.2)	162 (39.4)
Medications		
ACEi or ARB	399 (71.6)	235 (57.2)
Statins	345 (61.9)	176 (42.8)
Comorbidities		
Dementia	26 (4.7)	98 (23.8)
Cerebrovascular disease	54 (9.7)	68 (16.5)
Myocardial infarction	75 (13.5)	76 (18.5)
Congestive heart failure	138 (24.8)	167 (40.6)
Peripheral vascular disease	77 (13.8)	50 (12.2)
Chronic obstructive pulmonary disease	143 (25.7)	128 (31.1)
Mild liver disease	15 (2.7)	4 (1.0)
Moderate or severe liver disease	1 (0.2)	1 (0.2)
Peptic ulcer disease	29 (5.2)	27 (6.6)
Diabetes	302 (54.2)	216 (52.6)
Hypertension	538 (96.6)	382 (92.9)
Rheumatologic disease	13 (2.3)	10 (2.4)
Paraplegia/hemiplegia	10 (1.8)	4 (1.0)
Cancer	91 (16.3)	75 (18.2)
Metastatic solid tumor	6 (1.1)	13 (3.2)
Charlson-Deyo comorbidity index score ≥7	69 (12.4)	94 (22.9)

Abbreviations: ACEi, angiotensin-converting enzyme inhibitor; ARB, angiotensin-receptor blocker; eGFR, estimated glomerular filtration rate; IQR, interquartile range.

### Hospitalizations

**Figure 2.**
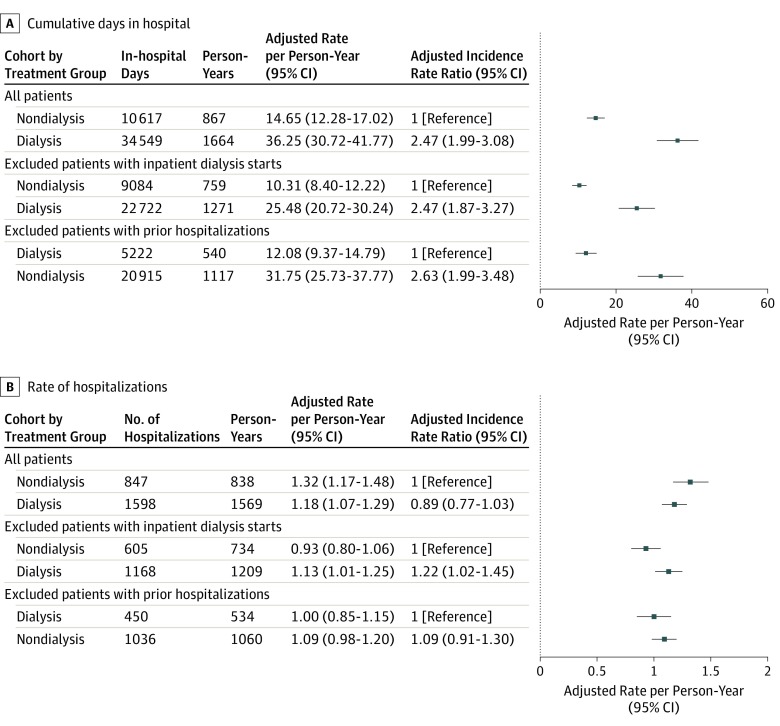
Adjusted Rates of Days Spent in the Hospital and Hospital Admissions Among Older Adults Treated vs Not Treated With Dialysis

Maintenance dialysis was associated with increased in-hospital days compared with nondialysis care. Overall, patients in the dialysis group spent an unadjusted rate of 35.29 (95% CI, 30.27-40.30) in-hospital days per person-year compared with 19.39 (95% CI, 16.47-22.31) in-hospital days per person-year in the nondialysis group; the adjusted IRR was 2.47 (95% CI, 1.99-3.08) (see Figure 2A and eTable 2 in the Supplement for unadjusted and adjusted rates). For a typical older patient in our cohort, treatment with maintenance dialysis would result in approximately 22 more in-hospital days per year than a patient not treated with dialysis. Dialysis was consistently associated with increased days spent in the hospital in sensitivity analyses excluding patients with previous hospitalizations (to examine patients with incident hospitalizations) (adjusted IRR, 2.63 [95% CI, 1.99-3.48]) and when excluding patients initiating dialysis in the hospital (IRR, 2.47 [95% CI, 1.87-3.27]).

Overall, however, dialysis was not associated with a higher rate of hospital admissions. The unadjusted rate of hospital admission for the dialysis group was 1.22 (95% CI, 1.10-1.33) hospital admissions per year compared with 1.44 (95% CI, 1.26-1.63) for the nondialysis group (adjusted IRR, 0.89 [95% CI, 0.77-1.03]) (see Figure 2B for adjusted rates). Results were consistent when we excluded patients with prior hospitalizations (adjusted IRR, 1.09 [95% CI, 0.91-1.30]). However, dialysis was associated with a higher rate of hospital admissions when excluding patients initiating dialysis in the hospital; the unadjusted rate was 1.09 (95% CI, 0.98-1.21) hospital admissions per year for dialysis and 1.06 (95% CI, 0.90-1.23) hospital admissions per year for nondialysis (adjusted IRR, 1.22 [95% CI, 1.02-1.45]) groups, because patients starting dialysis in the hospital had a high rate of hospitalizations after their index date but before their dialysis start date.

**Figure 3.**
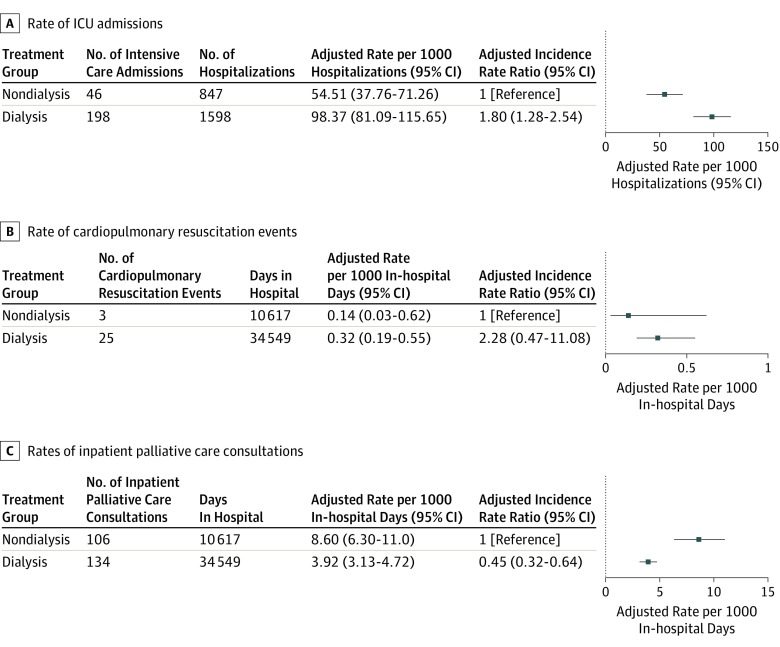
Adjusted Rates of Intensity of Care Among Older Adults Treated vs Not Treated With Dialysis ICU indicates intensive care unit.

Among patients who were hospitalized (n = 779), those treated with maintenance dialysis were more likely than those receiving nondialysis treatment to be admitted to an ICU (adjusted rate of ICU admissions per 1000 hospitalizations, 98.37 [95% CI, 81.09-115.65] vs 54.51 [95% CI, 37.76-71.26]; adjusted IRR, 1.80 [95% CI 1.28-2.54]) (see Figure 3A and eTable 2 in the Supplement for unadjusted and adjusted rates). There was no statistical demonstration of a difference between the groups for inpatient cardiopulmonary resuscitation (adjusted IRR, 2.28 [95% CI, 0.47-11.10]) (Figure 3B). However, patients in the dialysis group were less likely to receive inpatient palliative care (adjusted rate per 1000 in-hospital days, 3.92 [95% CI, 3.13-4.72] vs 8.60 [95% CI, 6.3-11.0]; adjusted IRR, 0.45 [95% CI, 0.32-0.64]) (Figure 3C). Among patients who died during follow-up (n = 672), a higher proportion of deaths occurred in a hospital setting among those treated with dialysis than those not treated with dialysis (unadjusted proportions, 221 [66.0%] vs 163 [48.4%]; adjusted proportions, 66.0% vs 24.3%; adjusted relative risk, 2.93 [95% CI, 2.51-3.41]).

### Other Outcomes

**Figure 4.**
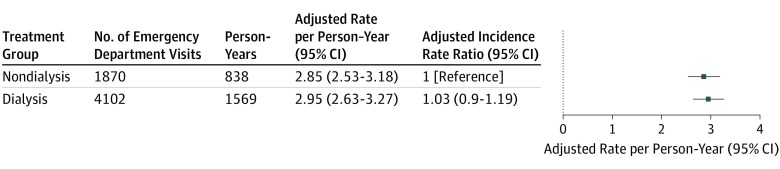
Adjusted Rates of Emergency Department Visits Among Older Adults Treated vs Not Treated With Dialysis

We did not observe an association between dialysis (vs nondialysis) care and rate of emergency department visits (unadjusted rate of 3.32 [95% CI, 2.86-3.77] per year for the dialysis group and 3.48 [95% CI, 3.01-3.94] per year for the nondialysis group; adjusted IRR, 1.03 [95% CI, 0.90-1.19]) (see Figure 4 and eTable 2 in Supplement for unadjusted and adjusted rates). Among patients with kidney failure not residing in a long-term care facility at the index date (n = 838), those treated with dialysis were as likely to be admitted to a long-term care facility as those receiving nondialysis treatment (adjusted hazard ratio, 1.11 [95% CI, 0.74-1.67]).

## Discussion

We found that older adults with kidney failure treated with maintenance dialysis spent more days in the hospital than those not treated with dialysis. According to this study, the magnitude of increased time in the hospital associated with maintenance dialysis may be clinically relevant: the average patient undergoing dialysis in our study spent an additional 22 in-hospital days per year compared with the otherwise similar patient who did not receive maintenance dialysis. However, the rate of hospital admissions was not higher. During their hospital stay, patients treated with dialysis had higher rates of admission to ICUs, similar rates of cardiopulmonary resuscitation, and lower rates of inpatient palliative care consultations. Patients undergoing dialysis were also more likely to die in a hospital setting. We found no differences between the dialysis and nondialysis groups in the rates of emergency department visits and likelihood of admission to long-term care.

Our findings are consistent with previous work in this field. In a nephrology-based clinic, Carson et al^20^ found that patients treated with dialysis vs nondialysis care had higher rates of hospital days. Carson et al^20^ and others^21,22^ also reported that patients receiving nondialysis treatment were more likely to die at home than those receiving dialysis. Morton et al^5^ demonstrated via a discrete-choice experiment that patients and caregivers across stages of chronic kidney disease (>50% patients with eGFR < 15 mL/min/1.73 m^2^, >40% aged ≥65 years) were willing to trade considerable life expectancy to reduce the burden and restrictions imposed by dialysis. Wong et al^23^ found that patients with sustained eGFR of less than 15 mL/min/1.73 m^2^ and not treated with dialysis during the final month of life received fewer intensive patterns of care, including hospitalization and receipt of an intensive procedure. During the final month of life, patients who decided to not pursue dialysis were also more likely to receive palliative and hospice care. Our study provides new information further quantifying the burden of dialysis in the Canadian setting not restricted to nephrology-referred patients.

Our observation that patients treated with maintenance dialysis spent more than 20 days per year in the hospital compared with those not treated with dialysis may be associated with dialysis-specific complications, structural issues, and overarching philosophy of care. Although most people would prefer to stay out of the hospital, hospitalizations are necessary when services are not accessible at home. In a multicenter prospective cohort in Canada, Quinn et al^24^ found that hospitalizations among patients receiving maintenance dialysis were most commonly owing to cardiovascular disease, infectious complications, and elective surgery, with almost 25% of hospital stays directly related to complications of dialysis or kidney disease. Further, patients undergoing dialysis may experience barriers to long-term care admission owing to their additional care needs, hence potentially prolonging their duration of stay in the hospital. Future research examining causes and opportunities to prevent prolonged hospitalization among patients receiving dialysis is warranted. Finally, maintenance dialysis may also be a proxy for the type of philosophy of care driving excess time in the hospital, intensive care, and less use of palliative care. Further exploration is required on other interventional possibilities, such as maintenance dialysis with a less aggressive philosophy of care, which would likely attenuate the apparent trade-offs associated with dialysis vs nondialysis care.

Among older adults living with medically complex circumstances, the decision to pursue dialysis or not is a preference-sensitive decision that aligns with a patient-centered paradigm.^25^ Although a previous study^3^ found that maintenance dialysis may reduce the risk of mortality within the first 3 years of kidney failure, this present study suggests that dialysis may be associated with an increase in time that patients spent in the hospital as well as receipt of more intensive health care services. From a patient perspective, future work should investigate prospective data and integrate estimates of survival, time spent in the hospital, and intensity of care in a personalized decision aid to communicate potential trade-offs and support shared treatment decision-making. From a policy perspective, our study identifies a need to plan for additional acute care resources for the growing aging population with kidney failure treated with dialysis globally.

Although more than 40% of older adults with kidney failure are treated without dialysis in Alberta, to our knowledge, the proportion of untreated patients in the United States and elsewhere is unknown. However, a prior study identified via medical record review that 14.5% of US patients with eGFR of less than 15mL/min/1.73 m^2^ in the Veterans Affairs setting made a decision not to pursue dialysis, and presumably a much larger percentage of patients in that cohort did not start dialysis during follow-up.^26^ Although universal provision is made for dialysis in Canada and the United States, the generalizability of our results to other settings in the United States and elsewhere is uncertain.

### Strengths and Limitations

Our study is strengthened by its population-based design in a setting with universal access to health care. We also used methodological rigor to reduce sources of bias (eg, treatment selection, lead time, and immortal time bias) found in prior studies that have examined similar outcomes between dialysis and nondialysis care groups. However, our study’s results must be interpreted in consideration of their limitations. First, our results are generalizable to a select cohort of older adults with creatinine measurements of less than 10 mL/min/1.73 m^2^ and relatively stable and slow progressive loss of eGFR, because we acknowledge that values can still fluctuate above and below low threshold eGFR values. Second, we assessed the cumulative length of hospital stay but did not have data on alternate level of care, hence limiting our ability to determine potential risk factors of prolonged length of hospital stay among dialysis patients. Third, although we were able to estimate the adjusted rates of admission to ICUs, we did not have access to the duration of time spent in ICUs. Fourth, we cannot exclude the possibility of residual confounding and were unable to account for potential confounders at baseline owing to lack of access to medical record data, which would provide more insight into the clinical context, including clinical stability and indication for dialysis initiation; these confounders include the symptoms or signs attributable to kidney failure (eg, pruritus), nutritional status, or frailty. Given our use of administrative data sources, we also did not have access to information on disease severity for most comorbidities; however, we were able to include severity of liver disease and kidney disease, identify clinically important demographic characteristics, and include a wide range of comorbidities and common medications. Fifth, we did not have information on patient preferences and therapeutic intent (ie, intent of conservative kidney management but started dialysis vs intent of dialysis care). Sixth, we a priori sought to examine patient outcomes spanning more than a decade (2002-2015) but acknowledge the broad era of inclusion and potential for changes over time in clinical practice regarding intensity and aggressiveness of end-of-life care for older adults with kidney failure. Finally, future work should examine outcomes in a prospective manner and other comparative data relevant to treatment decision-making for older adults with kidney failure and multiple comorbidities, including general health, symptom burden, physical and cognitive function, mental health, social participation, health-related quality of life, and caregiver burden.

## Conclusions

Although maintenance dialysis may decrease the risk of early mortality among older adults with kidney failure,^3^ we found that dialysis was associated with increased time spent in the hospital and receipt of intensive care. Therefore, it appears that dialysis may confer a trade-off between longer survival with more time in the hospital and ICU. These findings improve our understanding in patterns of health care use among a cohort of older adult patients with very advanced chronic kidney disease as a function of whether they initiated dialysis during follow-up, quantifying associated burdens of dialysis initiation and intensity of care.

## Notes

Supplement. **eTable 1.** Data Sources and *ICD* Codes for Rates of Inpatient Cardiopulmonary Resuscitation Events and Palliative Care Consultations **eTable 2.** Unadjusted and Adjusted Rates per Person-Year
