# Supplementary material for: Association of Initiation of Dialysis With Hospital Length of Stay and Intensity of Care in Older Adults With Kidney Failure
Source: JAMA Netw Open. 2020 Feb 28;3(2):e200222. doi: 10.1001/jamanetworkopen.2020.0222 (PMC7049084; doi:10.1001/jamanetworkopen.2020.0222)
Supplement: Supplement. — eTable 1. Data Sources and ICD Codes for Rates of Inpatient Cardiopulmonary Resuscitation Events and Palliative Care Consultations eTable 2. Unadjusted and Adjusted Rates per Person-Year [file jamanetwopen-3-e200222-s001.pdf]

Supplementary Online Content

Tam-Tham H, Ravani P, Zhang J, et al. Association of initiation of dialysis with hospital length of stay and intensity of care in older adults with kidney failure. *JAMA Netw Open*. 2020;3(2):e200222. doi:10.1001/jamanetworkopen.2020.0222

eTable 1. Data Sources and *ICD* Codes for Rates of Inpatient Cardiopulmonary Resuscitation Events and Palliative Care Consultations

eTable 2. Unadjusted and Adjusted Rates per Person-Year

This supplementary material has been provided by the authors to give readers additional information about their work.

©2020 Tam-Tham et al. *JAMA Network Open*.

eTable 1. Data Sources and *ICD* Codes for Rates of Inpatient Cardiopulmonary Resuscitation Events and Palliative Care Consultations

**Outcome**	**Source**	**ICD codes**
Rate of inpatient cardiopulmonary resuscitation events	Hospital Discharge Abstracts database	1HZ30 (1HZ30JN 1HZ30JY)<br>1HZ09 (1HZ09JAFS 1HZ09JAJF 1HZ09LACJ 1HZ09LAFS 1HZ09LAJF 1HZ09GRJF)
Rate of inpatient palliative care consultations	Hospital Discharge Abstracts database	Z515

Note: ICD=International Classification of Diseases; N/A=not applicable

©2020 Tam-Tham et al. *JAMA Network Open*.

eTable 2. Unadjusted and Adjusted Rates per Person-Year

Outcome	Cohort	Treatment group	No. of events	Observation time	Unadjusted rate	Unadjusted incidence rate ratio (95% CI)	Adjusted rate (95% CI)	Adjusted incidence rate ratio (95% CI)
			Days in hospital	Person-years	per person-year		per person-year	
Rate of days in hospital	All patients	Non-dialysis	10,617	867	19.39 (16.47-22.31)	Referent	14.65 (12.28-17.02)	Referent
		Dialysis	34,549	1,664	35.29 (30.27-40.30)	1.82 (1.50-2.21)	36.25 (30.72-41.77)	2.47 (1.99-3.08)
	Excluding inpatient dialysis starts	Non-dialysis	9,084	759	16.36 (13.49-19.23)	Referent	10.31 (8.40-12.22)	Referent
		Dialysis	22,722	1,271	22.24 (18.52-25.95)	1.36 (1.07-1.73)	25.48 (20.72-30.24)	2.47 (1.87-3.27)
	Excluding patients with prior hospitalizations	Non-dialysis	5,222	540	15.87 (12.35-19.39)	Referent	12.08 (9.37-14.79)	Referent
		Dialysis	20,915	1,117	31.53 (25.73-37.33)	1.98 (1.52-2.60)	31.75 (25.73-37.77)	2.63 (1.99-3.48)
			No. hospitalizations	Person-years	per person-year		per person-year	
Rate of hospitalizations	All patients	Non-dialysis	847	838	1.44 (1.26-1.63)	Referent	1.32 (1.17-1.48)	Referent
		Dialysis	1,598	1,569	1.22 (1.10-1.33)	0.84 (0.73-0.97)	1.18 (1.07-1.29)	0.89 (0.77-1.03)
	Excluding inpatient dialysis starts	Non-dialysis	605	734	1.06 (0.90-1.23)	Referent	0.93 (0.80-1.06)	Referent
		Dialysis	1,168	1,209	1.09 (0.98-1.21)	1.03 (0.86-1.22)	1.13 (1.01-1.25)	1.22 (1.02-1.45)
	Excluding patients with prior hospitalizations	Non-dialysis	450	534	1.09 (0.93-1.25)	Referent	1.00 (0.85-1.15)	Referent
		Dialysis	1,036	1,060	1.13 (0.99-1.26)	1.04 (0.88-1.22)	1.09 (0.98-1.20)	1.09 (0.91-1.30)
			No. ED visits	Person-years	per person-year		per person-year	
Rate of ED visits	Patients with hospitalizations	Non-dialysis	1,870	838	3.48 (3.01-3.94)	Referent	2.85 (2.53-3.18)	Referent
		Dialysis	4,102	1,569	3.32 (2.86-3.77)	0.95 (0.82-1.11)	2.95 (2.63-3.27)	1.03 (0.9-1.19)

©2020 Tam-Tham et al. *JAMA Network Open*.

Outcome	Cohort	Treatment group	No. of events	Observation time	Unadjusted rate	Unadjusted incidence rate ratio (95% CI)	Adjusted rate (95% CI)	Adjusted incidence rate ratio (95% CI)
			No. intensive care admissions	No. hospitalizations	per 1,000 hospitalizations		per 1,000 hospitalizations	
Rate of intensive care admissions	Patients with hospitalizations	Non-dialysis	46	847	55.32 (39.04-71.60)	Referent	54.51 (37.76-71.26)	Referent
		Dialysis	198	1,598	124.34 (105.66-143.01)	2.25 (1.63-3.10)	98.37 (81.09-115.65)	1.80 (1.28-2.54)
			No. CPR events	Days in hospital	per 1,000 in-hospital days		per 1,000 in-hospital days	
Rate of inpatient CPR events	Patients with hospitalizations	Non-dialysis	3	10,617	0.29 (0.09-0.96)	Referent	0.14 (0.03-0.62)	Referent
		Dialysis	25	34,549	0.88 (0.48-1.62)	1.23 (0.82-10.98)	0.32 (0.19-0.55)	2.28 (0.47-11.08)
			No. palliative care consults	Days in hospital	per 1,000 in-hospital days		per 1,000 in-hospital days	
Rate of inpatient palliative care consults	Patients with hospitalizations	Non-dialysis	106	10,617	10.0 (7.77-12.23)	Referent	8.6 (6.3-11.0)	Referent
		Dialysis	134	34,549	3.88 (3.26-4.60)	0.39 (0.29-0.52)	3.92 (3.13-4.72)	0.45 (0.32-0.64)

Note: ED=emergency department; CPR=cardiopulmonary resuscitation; No.=number; CI=confidence interval

©2020 Tam-Tham et al. *JAMA Network Open*.
